# The Proteasome Inhibitor CEP-18770 Induces Cell Death in Medulloblastoma

**DOI:** 10.3390/pharmaceutics16050672

**Published:** 2024-05-16

**Authors:** Swastina Nath Varma, Shany Ye, Sara Ferlin, Charley Comer, Kian Cotton, Maria Victoria Niklison-Chirou

**Affiliations:** 1 Blizard Institute, Queen Mary University of London, London E1 4NS, UK; t.varma@ucl.ac.uk; 2 Life Sciences Department, University of Bath, Claverton Down, Bath BA2 7AY, UK; sy996@bath.ac.uk (S.Y.); slf49@bath.ac.uk (S.F.); cc2736@bath.ac.uk (C.C.); kc691@bath.ac.uk (K.C.)

**Keywords:** medulloblastoma, CEP-18770, cancer, proteasome inhibitors, brain tumors

## Abstract

Medulloblastomas (MBs) represent the most prevalent malignant solid tumors in kids. The conventional treatment regimen for MBs includes surgical removal of the tumor, followed by radiation and chemotherapy. However, this approach is associated with significant morbidity and detrimental side effects. Consequently, there is a critical demand for more precise and less harmful treatments to enhance the quality of life for survivors. CEP-18770, a novel proteasome inhibitor that targets the 20S subunit, has emerged as a promising candidate, due to its anticancer activity in metastatic solid tumors and multiple myeloma, coupled with an acceptable safety profile. In this study, we aimed to assess the anticancer efficacy of CEP-18770 by employing a variety of MB patient-derived cells and cell lines. Our preclinical investigations revealed that CEP-18770 effectively inhibits proteasome activity and induces apoptosis in MBs cells. Furthermore, we discovered that CEP-18770 and cisplatin, a current component of MB therapy, exhibit a synergistic apoptotic effect. This paper shows that CEP-18770 holds potential as an adjunctive treatment for MB tumors, thereby paving the way for more targeted and less toxic therapeutic strategies.

## 1. Introduction

Medulloblastomas (MBs) are aggressive brain tumors commonly found in children, characterized by high metastatic potential (WHO grade IV) [1,2]. These embryonal tumors originate in the cerebellum and are believed to stem from distinct neuronal stem or progenitor cell populations during early life [2]. Aggressive MBs have a tendency to spread to other parts of the brain via the cerebrospinal fluid or the blood, making treatment very difficult [3]. This disease predominantly affects children, where 70% of the cases occur in patients 16 years old or younger, with a peak incidence occurring at age seven [3]. The survival rates of affected children have seen improvement recently, thanks to the current standard of treatment which involves surgery, followed by chemotherapy (cisplatin, etoposide and vincristine) and high-dose craniospinal irradiation. However, despite the efficacy in targeting the tumor, these treatments have substantial drawbacks, as survivors often endure considerable side effects, such as long-term neurocognitive, endocrine, and other toxicities [4]. Therefore, new treatments targeting MB metastases in particular are crucial, to minimize the adverse effects associated with existing therapies. MBs have been classified into four distinct molecular subgroups named Wingless (WNT—good prognosis), Sonic Hedgehog (SHH—intermediate prognosis), Group 3 (G3—worst prognosis), and Group 4 (G4—intermediate prognosis) [5]. G3-MB and G4-MB are the most aggressive and least characterized of all subgroups. They predominantly occur in young children and are associated with MYC or MYCN amplification. However, p53, a tumor suppressor protein commonly mutated in various cancers, is never mutated in aggressive MBs [6]. Importantly, we recently reported that G3-MB and G4-MB express high levels of p73, a member of the p53-family [7]. In these tumors, p73 sustains cell proliferation by activating glutamine metabolism [X]. Cisplatin is among the chemotherapeutic drugs used in the treatment of MBs and is known to activate p53 and p73, which has been reported to activate transcription of pro-apoptotic genes [8]. Whilst the p53-family members are regulated at the level of gene expression, their protein levels are also controlled through the ubiquitin–proteasome pathway. The ubiquitin–proteasome pathway (UPP) has an essential role in maintaining the homeostasis of intracellular proteins. This multi-enzymatic complex is responsible for the degradation of proteins that are damaged, mutant, or those that must be maintained at low levels such as pro-apoptotic proteins like the p53-family [9]. The first step in the UPP involves the marking of unwanted proteins with ubiquitin molecules. Targeted proteins are recognized by the 26S proteasome complex, composed of the catalytic 20S core subunit and the two terminal regulatory particles known as 19S proteasome [10]. Low activity of the UPP has been implicated in neurodegenerative diseases such as Alzheimer’s and Parkinson’s [11]. On the other hand, increased proteasome activity has been extensively demonstrated in cancer [12,13]. Moreover, previous studies from the lab have shown that MB cells upregulate the proteasome pathway. We observed substantial upregulation of all 19S (PMSD and PMSC) and 20S proteasome (PMSA and PMSB) subunits in MB cells [14]. The use of proteasome inhibitors therefore appears to be a promising avenue of treatment for MBs.

Proteasome inhibitors (PIs) are drugs that either reversibly or irreversibly block the activity of the 26S proteasome complex. The first FDA approved PI was bortezomib, and it is available on the market for the treatment of some cancers such as leukemia and multiple myeloma [12,14]. CEP-18770 is a novel PI that produces a significant decrease in tumor size and has a dose-related incidence of complete tumor regression with minimal changes in animal body weight over the course of 120-day treatment [12,15,16]. When compared to bortezomib, equivalent doses of CEP-18770 show a greater and more sustained dose-related inhibition of tumor proteasome activity, corresponding temporarily with maximum induction of apoptosis [12]. No proteasome inhibition was detected in normal mouse brain tissue at any time point for either CEP-18770 or bortezomib, indicating that these PIs cannot cross the blood–brain barrier (BBB) [17]. Importantly, tumors compromise the integrity of the BBB, leading to the formation of what is referred to as the blood–tumor barrier. This barrier is notably heterogeneous and exhibits unique characteristics, such as an uneven permeability and the active expulsion of molecules [18]. Consequently, it may be feasible for CEP-18770 to penetrate the blood–tumor barrier.

In this study, we sought to investigate the effect of CEP-18770 on MB cells. First, we show that four PIs (MG-132, Carfilzomib, CEP-18770 and NPI-0052) reduced the cell viability of MB cells. We also demonstrate that aggressive and non-aggressive MBs cells are sensitive to CEP-18770. Remarkably, we show that the p53 family plays a role in CEP-18770’s mechanism of action by increasing reactive oxygen species (ROS) levels. Notably, CEP-18770 has a synergistic effect with cisplatin, a current component of the standard MB patient therapy. These findings support the possibility that CEP-18770 could be used as an adjuvant treatment for the most aggressive and invasive MBs tumors.

## 2. Materials and Methods

### 2.1. Chemicals

The chemicals used for the experiments were CEP-18770 and ciplastin. Ciplastin was purchased from Sigma-Aldrich (St. Louis, MO, USA) and CEP-18770 was obtained from BioVison Incorporated, Milpitas, CA, USA.

CEP-18770 was diluted in 100% dimethyl sulfoxide (DMSO) to prepare the stock solution. The stock solutions were further diluted in a 1:10 serial dilution with phosphate-buffered saline (PBS) to different concentrations.

### 2.2. Cell Culture

Four different medulloblastoma cell lines were used for the experiments: DAOY, UW228-2, D-425, and D-458. DAOY and UW228-2 were obtained from ATCC. D-425 and D-458 were obtained from Dr Silvia Marino, Queen Mary University of London. The medulloblastoma cell lines were cultured in DMEM + GlutaMAX™ (Gibco^®^, Grand Island, NY, USA) media supplemented with 10% fetal bovine serum (FBS), 1% penicillin, and streptomycin and 1% amino acids. All cell lines were incubated at 37 °C in 5% CO_2_ humidified air.

### 2.3. Knockdown of p73

Small interfering RNA (siRNA) constructs targeting p73 (sip73*3: ID: 2671; sip73*5: ID: 115666) and a non-targeting control siRNA (scramble) were purchased from Ambion. DAOY cells were transfected with 10 pM siRNA with Lipofectamine 3000 (Life Technologies, Frederick, MD, USA) according to the supplier’s protocol.

### 2.4. Drug Treatment of the Cell Lines

All medulloblastoma cells were counted before plating using a CountessTM 3 FL Automated cell counter (Thermo Fisher Scientific, Waltham, MA, USA). Before counting, DAOY and UW228-2 were dissociated with 1% trypsin. After the cells had detached, DMEM + GlutaMAX™ (Gibco^®^) media was added to neutralize the trypsin. A total volume of 6 mL was transferred to a 50 mL Falcon tube. From the Falcon tube 20 μL cell suspension was mixed with 20 μL trypan blue in an Eppendorf, and 10 μL of the mixture was pipetted into a Countess chamber slide.

The cell suspension of the D-425 and D-458 cell lines was transferred to a 50 mL Falcon tube and centrifuged for 5 min at 500 rpm. The media were removed, and the cell pellets were resuspended with 2 mL of new DMEM + GlutaMAX™ (Gibco^®^) media. From the Falcon tube, 20 μL cell suspensions were mixed with 20 μL trypan blue in an Eppendorf and 10 μL of the mixtures was pipetted into a Countess chamber slide.

After counting the cells, between 5000 and 10,000 cells were plated in each well of a 96-well plate with a total volume of 100 μL. The 96-well plates were incubated for 24 h before treatment. After 24 h of incubation, all cells were treated with different drug concentrations of CEP-18700. Controls were treated with an equal volume of media. The treatment lasted for 48 h, before the cell viabilities were quantified using a CellTiter-Glo^®^ (Promega, Madison, WI, USA) luminescent cell viability assay.

### 2.5. In Vitro 3D Models—Spheroids

DAOY and UW228-2 cell lines were counted by using a CountessTM 3 FL Automated cell counter (Thermo Fisher Scientific, Waltham, MA, USA). Before counting, the cell lines were dissociated with 1% trypsin; after the cells had detached, DMEM + GlutaMAX™ (Gibco^®^) media were added to neutralize trypsin. After counting the cells, volumes of cell suspensions having 500 or 1000 cells of DAOY and UW228-2, respectively, in 66 wells were transferred to a 15 mL Falcon tube and centrifuged at 1000 rpm for 5 min. The supernatant was discarded, and the cell pellets were resuspended in 13.2 mL neurosphere media. To the outer layer of a U round-bottom 96-well plate, 200 μL of sterile PBS was added. For the remaining wells, a total volume of 200 μL of cell suspensions was added. The U round-bottom 96-well plates were incubated for four days before treatment of the spheroids started.

After four days of incubation, 100 μL of media was removed and replaced with 100 μL new neurosphere media. Spheroids were then treated with different drug concentrations of CEP-18700. The treatment lasted between 11 and 14 days before the cell viabilities were quantified using a CellTiter-Glo^®^ luminescent cell viability assay. An EVOS M5000 imaging system (Thermo Fisher Scientific, Waltham, MA, USA) was used to measure the size and to take brightfield images of the spheroids before and during treatment with CEP-18770.

### 2.6. Cell Viability

Cell viability was quantified by CellTiter-Glo^®^ luminescent cell viability assay. Detection was based on using the luciferase reaction to measure the amount of ATP from viable cells. The amount of ATP in cells correlated with cell viability. This was performed using the Luciferin/Luciferase method with the help of a luminosity measuring plate reader. The plate used was opaque to limit natural light interference. The intensity of the emitted light due to the degradation of D-Luciferin and ATP by the enzyme Luciferase is proportional to the amount of free ATP present in the cells at that moment. Cell viability analysis was obtained by luminescence measurement via the GEN 5 plate reader.

### 2.7. Apoptosis Assay by Flow Cytometry

Flow cytometry was performed in order to measure the levels of apoptosis. In this assay, we detected the externalization of phosphatidylserine in apoptotic cells using recombinant annexin V conjugated to green-fluorescent FITC dye and dead cells using propidium iodide (PI). PI stains necrotic cells with red fluorescence. After treatment with both probes, apoptotic cells show green fluorescence, dead cells show red and green fluorescence, and live cells show little or no fluorescence.

The MB cells were dissociated with trypsin and then washed in binding buffer. Annexin V (25 ug/mL) was then added to the cell suspension. The cells were then left to sit in the FACS tubes for 10 min in darkness at room temperature. PI (1 ug/mL) staining solution was then added and FACS was carried out.

### 2.8. Western Blotting Analysis

MB cells were dissociated with trypsin and then sonicated for 5 min in RIPA buffer (50 mM Tris–HCl (pH 7.5), 150 NaCl, 1% NP-40, 0.25% sodium deoxycholate). Proteins were quantified using a Pierce™ Coomassie (Bradford, UK) protein assay kit and each sample was separated by SDS-PAGE (8% polyacrylamide gels). Proteins were transferred onto nitrocellulose membranes, which were then blocked in 5% non-fat milk diluted in Tris buffered saline (TBS) containing 0.5% Tween 20 (TBS-T) for 30 min at room temperature. Primary antibodies were diluted in 5% non-fat milk in TBS-T and incubated at 4 °C overnight. We used rabbit anti-p73 (1/1000) (polyclonal antibody, A300-126A, Bethyl Laboratories Inc., Montgomery, TX, USA) and rabbit anti-Ubiquitin P4D1 (1/1000) (monoclonal antibody, Cell Signaling Technologies, Danvers, MA, USA). Mouse anti-α Tubulin (1/5000) (SIGMA) was used as a loading control.

The membranes were washed three times for 10 min in TBS-T. Secondary antibodies anti-mouse (1/10,000) or anti-rabbit (1/10,000) were diluted in 5% non-fat milk in TBS-T and incubated for 1 h in the dark at room temperature. The membranes were washed in TBS-T three times for 10 min. The membranes were then incubated in Lumi-Light Western blotting substrate (Roche Diagnostics, Indianapolis, IN, USA). The membranes were exposed to film and developed.

### 2.9. Protein Quantification

To ensure that the same amount of protein was added to each well in the WB gel, the protein had to be quantified. This was performed using a Pierce™ Assay Kit: when the reagent mixture is added to a protein sample (2 μL), it changes color from brown to blue in proportion to how much protein is present. Samples were incubated at 37 °C for 5 min and were measured for absorbance at the optimal wavelength, 595 nm, using a GEN 5 plate reader and software. We used a universally accepted reference protein for total protein quantitation, known as BSA solution, as a standard.

### 2.10. Immunofluorescence (IF)

Glass coverslips were sterilized with 100% ethanol. DAOY or UW228-2 cells were then grown on these sterilized coverslips. Cells were treated with different concentrations of cisplatin and cisplatin plus CEP-18870. After 24 h, the cells were fixed using 4% paraformaldehyde in PBS pH 7.4 for 10 min at room temperature. If the target protein is intracellular, it is very important to permeabilize the cells. The samples were incubated for 10 min with PBS containing either 0.1% or 0.25% Triton X-100. The cells were then washed three times with PBS for 5 min each.

Cells were incubated with 10% goat serum. The cells were then incubated with anti-γH2AX (Cell Signaling/Abcam) (1/250) in 1% BSA in TBS-T in a humidified chamber overnight at 4 °C. The solution was decanted and the cells were washed three times in PBS for 5 min each. The cells were incubated with 0.1–1 μg/mL DAPI (DNA stain in blue) for 5 min before being rinsed with PBS. The cells were incubated with the secondary antibody anti-Rabbit Alexa Fluor^®^ 488 (green) (1/1000) in 1% BSA for 1 h at room temperature in the dark. The secondary antibody solution was decanted and washed three times with PBS for 5 min each in the dark. Each coverslip was mounted with a drop of mounting medium and sealed with nail polish to prevent drying and movement under the microscope.

Images were taken in an epifluorescence or confocal microscope.

### 2.11. Statistical Analysis

All results are expressed as mean values ±SD or ±SEM of at least three independent experiments. An unpaired Student t test was used to generate statistical analyses. *P* values <0.05 were considered statistically significant.

## 3. Results

Proteasome inhibitors reduced cell viability of aggressive and non-aggressive MBs cells.

**Figure 1:**
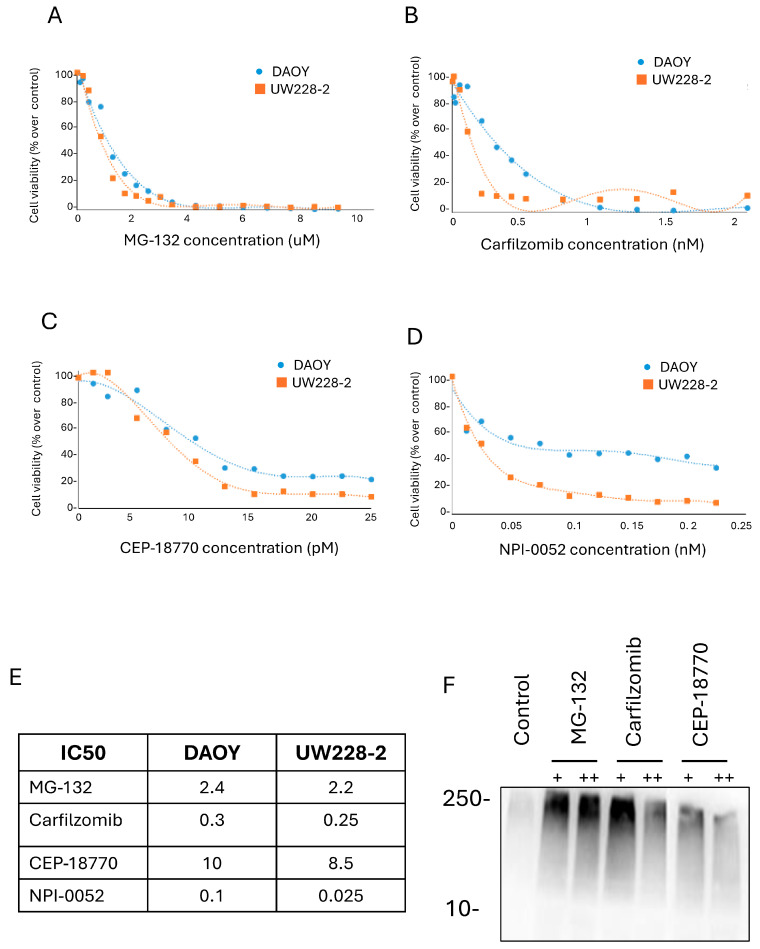
Proteasome inhibitors significantly reduced MB cells viability. Representative IC50 fitting curves of MG-132, Carfilzomib, CEP-18770, and NPI-0052. (**A**–**D**) DAOY and UW228-2 cells were treated for 48 h with different concentrations of MG-132 (**A**), Carfilzomib (**B**), CEP-18770 (**C**), and NPI-0052 (**D**). (**E**) Cell viability was determined by Cell TiterGlo assay and the IC50 was calculated with a non-linear regression curve. (**F**) Western blotting of polyubiquitin protein in DAOY cells after treatment with two concentrations of MG-132, Carfilzomib, and CEP-18770 each. Polyubiquitin was detected by anti-ubiquitin antibody on the total lysate of the cells. +: 1.2 uM MG-132, 0.125 nM Carfilzomib, 7.5 pM CEP-18770. ++: 2.4 uM MG-132, 0.25 nM Carfilzomib, 15 pM CEP-18770.

Proteasome inhibitors (PIs) are novel and potent anti-cancer drugs [12,14,19]. We first tested the effect of three novel PIs (Carfilzomib, CEP-18770, and NPI-0052) and one research PI (MG-132) in non-aggressive MB cells. Titration curves were performed by adding increased concentrations of PIs, and viability was measured after 48 h of treatment. PIs reduced cell viability in DAOY and UW228-2 cells in a concentration-dependent manner (Figure 1A–D). Following this, non-linear regression was calculated to estimate the IC50 (Figure 1A–D). These results are represented in the IC50 table (Figure 1E), showing that the strongest PI was CEP-18870, followed by Carfilzomib and NPI-0052, which had an intermedial effect, and the weakest PI was MG-132, which was used as a positive control, as it is not used in clinical settings due to high toxicity.

Following this, to evaluate if aggressive and non-aggressive MB cells had different sensitivity to PIs, we repeated the experiment with G3-MBs cells (D-425 and D-458) alongside the non-aggressive cells (DAOY and UW228-2). Interestingly, all MBs cells showed equal sensitivity to CEP-18770 and NPI-0052 (Supplementary Figure S1A,B).

Upon treatment with PIs, cells begin to accumulate proteins tagged with Ub [20]. We sought to confirm whether the treatment of MG-132, Carfilzomib, and CEP-18770 on DAOY cells induced the accumulation of ubiquitylated proteins by performing a Western blot with an anti-ubiquitin antibody. Western blot of Ub proteins typically appears as a high molecular weight smear caused by heterogeneity of the modified proteins (Figure 1F). MG-132, Carfilzomib, and CEP-18770 induced the accumulation of polyubiquitin proteins, resulting in an increased smear of protein in comparison to the control.

These results show that novel PIs are very effective in reducing cell viability of MBs.

Pre-treatment with cisplatin followed by CEP-18770 has a synergistic effect on cell death in MB cell lines.

As mentioned, the drugs used as standard treatment for MB tumors primarily include vincristine, cisplatin, and etoposide [21]. To study the effect of cisplatin and etoposide in DAOY and UW228-2, we performed a titration curve. Different concentrations of cisplatin and etoposide were added to DAOY and UW228-2 cells for 48 h, after which cell viability was measured. Supplementary Figure S2 shows the representative IC50 fitting curves for cisplatin and etoposide in DAOY and UW228-2. As expected, increasing concentrations of cisplatin and etoposide reduced the viability of DAOY cells (Supplementary Figure S2A,B). Interestingly, UW228-2 cells were significantly more resistant to cisplatin and etoposide treatment than DAOY (Supplementary Figure S2A,B).

**Figure 2:**
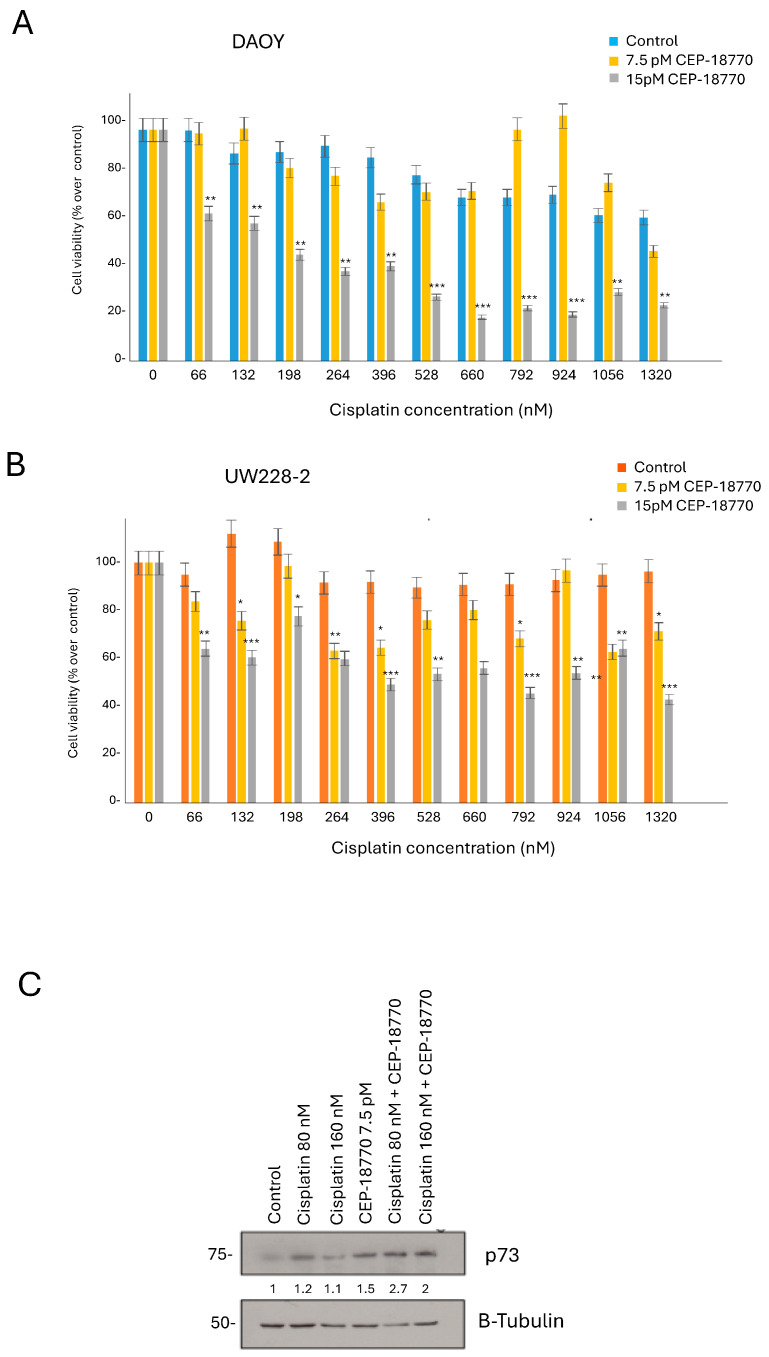
Synergistic effect between cisplatin pre-treatment and CEP-18770 in MBs cells. (**A**) DAOY and (**B**) UW228-2 cells were treated with different concentrations of cisplatin first, and after 10 h CEP-18770 was added. We tested two concentrations of CEP-18770, 7.5 pM and 15 pM. The cells were incubated for a further 30 h and cell viability was determined by Cell TiterGlo assay. (**C**) Western blot showing p73 levels in DAOY cells after cisplatin pre-treatment and 7.5 pM CEP-18770 was added. Two concentrations of cisplatin were tested: 80 and 160 nM. Tubulin was used as a loading control. * *p* < 0.05, ** *p* < 0.001; *** *p* < 0.0001.

As pre-treatment with different drugs to sensitize aggressive cancers is a common practice [22], we decided to test if the pre-treatment of MB cells with cisplatin would sensitize them to CEP-18770 treatment. We decided to investigate the effect of CEP-18770 alone, as it was the strongest PI with our cells. We performed a titration experiment where DAOY and UW228-2 cells received different concentrations of cisplatin first, and 10 h later a single dose of either 7.5 pM or 15 pM CEP-18770 (Figure 2A,B). We observed that 7.5 pM or 15 pM CEP-18770 alone did not reduce cell viability. However, the combination of cisplatin plus CEP-18770 reduced cell viability in a cisplatin-concentration-dependent manner. These results demonstrate that there is a strong synergistic effect in reducing cell viability between the cisplatin pre-treatment and CEP-18770 combination in DAOY and UW228-2 cells (Figure 2A,B).

We hypothesized that this cell death effect was driven by p73, a tumor suppressor protein that induces apoptosis in cells. This member of the p53-family is essential for brain development, but its precise role remains unclear in brain tumors. p73 is never mutated in cancer cells, and therefore we measured p73 levels in DAOY cells, which harbor p53-mutant protein [14], after cisplatin treatment alone, CEP-18770 alone, and cisplatin pre-treatment followed by CEP-18770. Importantly, all of our treatments induced p73 stabilization, but the effect was stronger in the combination treatment, cisplatin plus CEP-18770 (Figure 2C). Next, we set out to assess the impact of p73 silencing on MB cells. Indeed, knockdown of p73 in DAOY cells (Supplementary Figure S2C,D) significantly rescued the cell viability after cisplatin treatment.

These results demonstrate that cisplatin pre-treatment and CEP-18770 combination reduces cell viability in DAOY and UW228-2 cells, possibly through a p73-dependent mechanism.

Cisplatin pre-treatment causes DNA damage that is enhanced by CEP-18770 treatment.

It was reported that cisplatin treatment induced ROS that could induce DNA damage [23,24], and it has been well documented that p73 is stabilized after DNA damage [25,26]. Therefore, we decided to test the hypothesis that the synergic effect in cell viability seen following cisplatin pre-treatment and CEP-18770 combination could be due to an increase in DNA damage.

**Figure 3:**
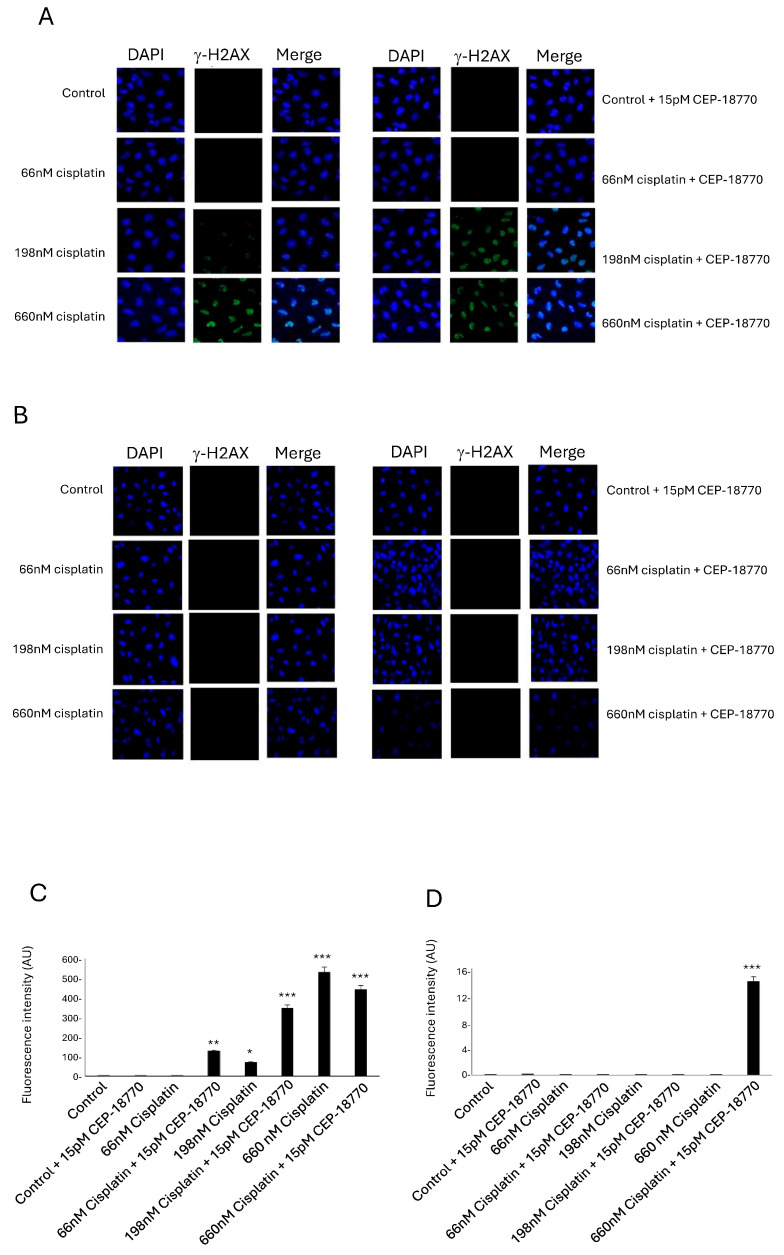
Cisplatin pre-treatment follow by CEP-18770 induced DNA damage in MB cells. (**A**,**C**) DAOY and (**B**,**D**) UW228-2 cells were treated with different concentrations of cisplatin 66, 198 and 660 nM, and after 10 h CEP-18770 was added to the cells. Following this, the cells were fixed with 4% PFA and stained for γH2AX. (**A**,**B**) Representative images of the γ-H2AX staining were taken with a 10× objective. (**C**,**D**) Quantification of total fluorescence of γ-H2AX was performed using ImageJ, and statistical analysis was performed using one-way ANOVA. At least 300 cells were counted per experiment. The experiment was repeated three times. * *p* < 0.05; ** *p* < 0.01; *** *p* < 0.001.

We pre-treated DAOY and UW228-2 cells with different concentrations of cisplatin, and after 10 h, CEP-18770 was added to the cells. We detected DNA damage with the probe γH2AX. γH2AX accumulates in the nucleus of cells and forms foci at sites of DNA double-strand breaks [27]. Importantly, high concentrations of cisplatin successfully induced DNA damage, but this damage was increased following the addition of CEP-18770 (Figure 3A,C). However, UW228-2 cells did not show DNA damage after cisplatin treatment at this dose (Figure 3B,D).

These results confirmed our hypothesis that pre-treatment with cisplatin induces oxidative stress in MB cells and that this effect is enhanced when CEP-18770 is added. Based on the fact that the UPP is involved in a variety of cellular processes, including DNA repair, transcriptional regulation, signal transduction, and cell metabolism [20], we hypothesized that CEP-18770 has a strong effect on the inhibition of DNA repair proteins, causing DNA damage to accumulate in the cells. Our results show that cells expressing stable p73 are more sensitive to DNA damage.

Apoptotic effect of cisplatin plus CEP-18770 in DAOY cells.

**Figure 4:**
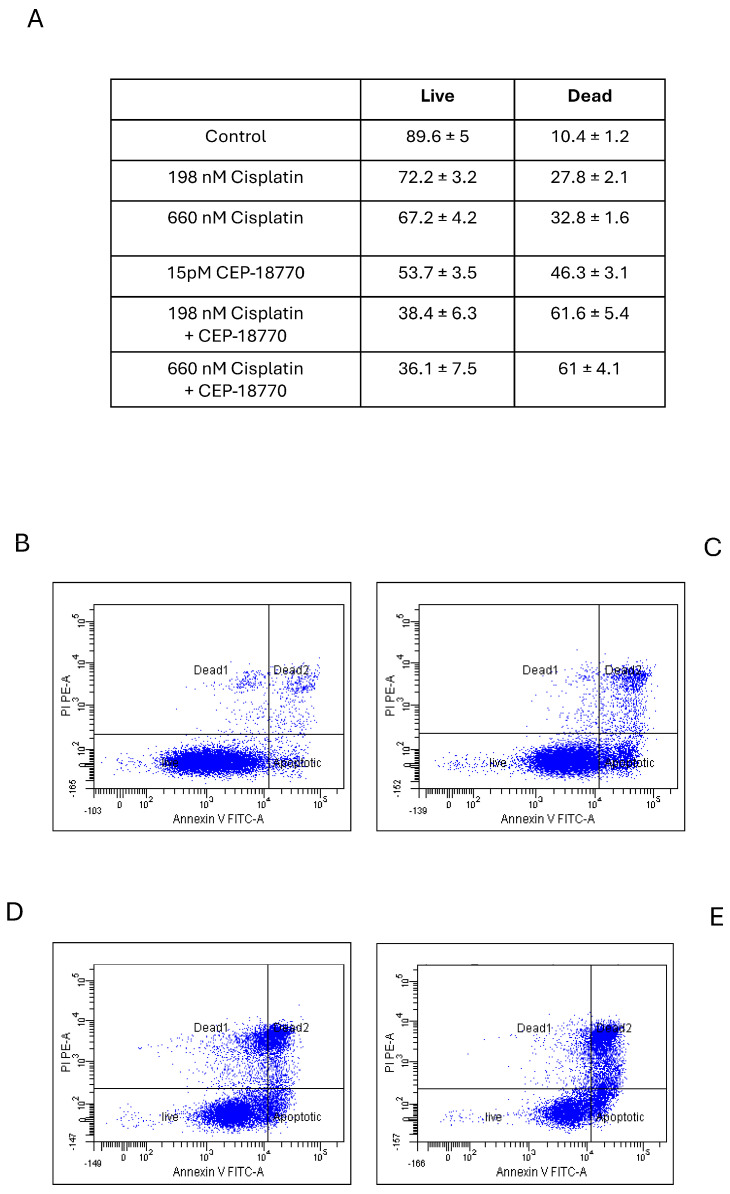
Cisplatin pre-treatment and CEP-18770 induced apoptosis in DAOY cells. DAOY cells were treated with different concentrations of cisplatin 198 and 660 nM, and after 10 h CEP-18770 was added to the cells. After 48 h, the cells were collected and stained for Annexin V/PI. FACS analysis was performed and cells death was measured. (**A**) Quantification of cell death and alive. (**B**–**E**) Representative FACS analysis images for (**B**) control, (**C**) 660 nM cisplatin, (**D**) 15pM CEP-18770, and (**E**) CEP-18770 + cisplatin. Statistical analysis was performed using one-way ANOVA. The experiment was repeated three times.

We then decided to investigate whether the reduction in cell viability was due to apoptosis induction. Therefore, we measured apoptosis by Annexin V/PI analysis. DAOY cells were pre-treated with cisplatin, and after 10 h CEP-18770 was added to the cells for 30 h. Cells were collected and apoptosis was measured. Figure 4A shows that increasing concentrations of cisplatin caused an increase in apoptosis. More importantly, CEP-18770 also induced cell death. Figure 4B–E displays representative images of flow cytometry readings that show strong cell death after the combination treatment of CEP-18770 and cisplatin.

CEP-18770 reduces cell viability in 3D MB culture.

**Figure 5:**
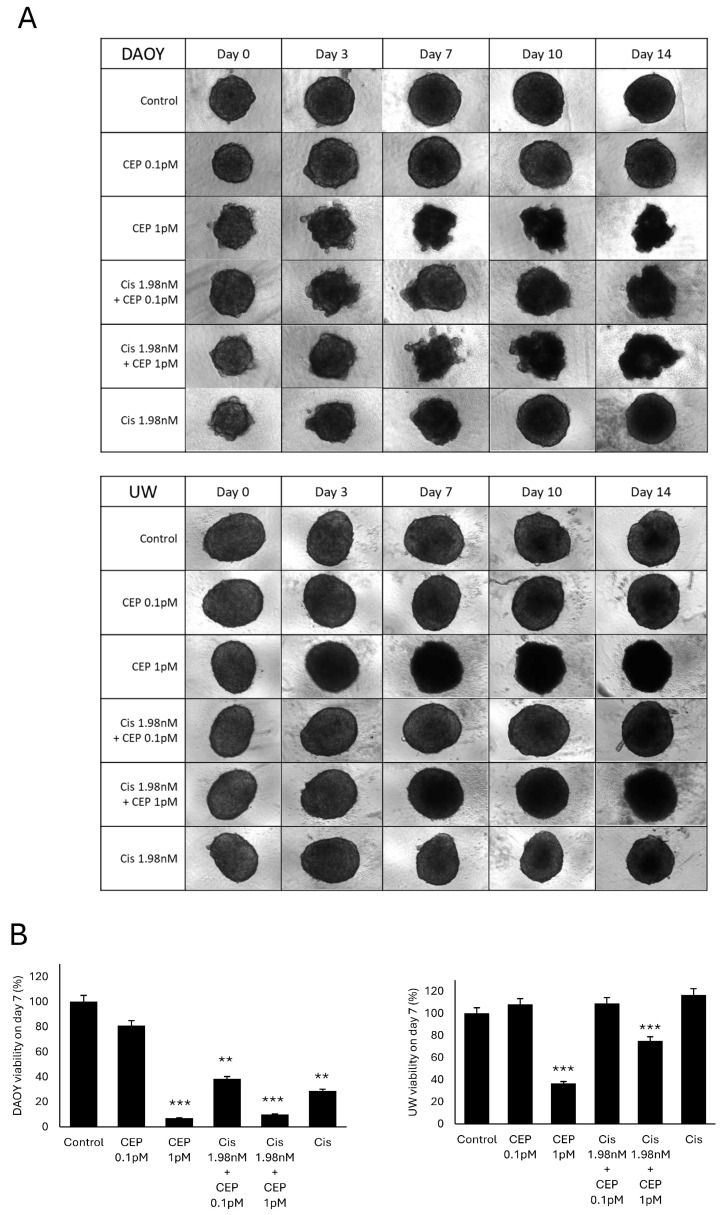
CEP-18770 induced cell death of MBs spheroids. DAOY and UW228-2 cells were grown as spheroids, and after 4 days cells were treated with CEP-18770, cisplatin, or a combination of CEP-18770 plus cisplatin. Cell medium was changed every 2 or 3 days and fresh 0.1 or 1 pM CEP-18770 or 1.98 nM cisplatin was added. (**A**) Representative images of DAOY and UW228-2 spheroids after 0, 3, 7, 10, and 14 days of treatment with CEP-18770, cisplatin, or a combination of CEP-18770 and cisplatin. (**B**) After 7 days of treatment, cell viability was measured with Cell TiterGlo. The results are presented as mean value of the cell viability over control. ** *p* < 0.01; *** *p* < 0.001.

Using 3D models has been an incredibly useful tool to determine drug responses of cancer cells in recent years [28]. Three-dimensional models of MB cells can provide a more accurate representation of tumor characteristics such as metabolism, drug response, and migration [28]. Two medulloblastoma cell lines (DAOY and UW228-2) were grown as spheres. The diameter of the spheroid was measured, and values are shown in Figure 5A and Supplementary Figure S3A,B. We initially observed that control cells grew naturally over time. At day 4, spheroids were sequentially treated with low doses of CEP-18770 (Figure 5A). To quantify the response to CEP-18770 treatment, cell viability was measured at day 7 of treatment. Figure 5B shows that CEP-18770 significantly reduced the cell viability in the 3D model of the medulloblastoma cell lines, both in DAOY and UW228-2. All concentrations of CEP-18770 significantly reduced MB cell viability and induced a strong apoptotic effect. Subsequently, we performed combination experiments between CEP-18770 and cisplatin. We did not observe a synergistic effect in any of the MB cells. Importantly, MBs that were resistant to cisplatin treatment were still sensible to CEP-18700 treatment. These data indicate that CEP-18770 is a promising drug to sensitize MBs tumors.

## 4. Conclusions

Medulloblastomas are the most common type of brain tumor in children [1]. Current treatment includes a combination of surgery, radiotherapy, and chemotherapy, leaving survivors with severe side effects. Therefore, new therapies that are more targeted and less toxic are crucial for improving the quality of life of survivors. It was recently reported that p73, a member of the p53 family, is overexpressed in MBs tumors. p73 is important for brain development and inducing apoptosis after DNA damage. Importantly, p73 levels are regulated by degradation in the UPP via the 26S proteasome [8,20,29,30]. The UPP plays a critical role in regulating many processes in the cell that are important for tumor cell growth and survival [31]. Inhibition of the proteasome function has emerged as a powerful strategy for anti-cancer therapy [20]. Importantly, second-generation PIs have been developed with improved pharmacological properties and less toxicity [32].

Of importance to MBs, some of these new PIs are able to cross the BBB [19]. In this work, we confirmed that p73 was overexpressed in human MBs cell lines. These data are in agreement with previous studies showing high p73 levels in MBs cells [8,33]. As p73 levels are regulated by degradation via the proteasome, we hypothesized that cells with high levels of p73 will be more sensitive to PI treatment. All of the PIs act by targeting the UPP and inhibiting the 26S proteasome.

We then selected cisplatin and etoposide as chemotherapeutic agents to treat MB cells, because they are currently used to treat children with MBs [21]. Cisplatin and etoposide have two very different mechanisms of action. Cisplatin induces cell cycle arrest and apoptosis by regulating the activation of several signal transduction pathways. One of the most important mechanisms of cisplatin is the induction of ROS generation and thus oxidative stress, especially in the mitochondria, to activate cell death pathways. Cisplatin can also cause DNA damage that prevents cell division and causes apoptosis [23,24]. On the other hand, etoposide prevents cell cycle progression and DNA repair by targeting DNA topoisomerases, enzymes that regulate genetic material by causing temporary breaks in DNA [34,35]. Cisplatin and etoposide are known to be a very active drug combination when given before radiation [36]. In contrast to DAOY, when we added cisplatin and etoposide to UW228-2 cells, we observed no change in cell viability. The differences in DAOY and UW228-2 results could be due to the expression of p73, as DAOY expresses higher levels of p73.

We pre-treated cells before testing different PIs in all of the subsequent experiments. We considered whether the different PIs could sensitize MB cells to cisplatin treatment. We observed that cisplatin pre-treatment and CEP-18770 was the only treatment that induced a synergic effect in reducing cell viability in both DAOY and UW228-2 cells. However, the synergic effect of reducing cell viability was stronger in DAOY cells; again, perhaps due to the presence of p73 in this cell line.

It is well documented that cisplatin induces ROS, which can induce DNA damage [23,24]. As p73 synthesis is activated after DNA damage [23], we hypothesized that this effect was responsible for the response of the DAOY cells and this effect would be enhanced by further treatment with CEP-18770. To validate this hypothesis, we performed an immunofluorescence assay in DAOY and UW228-2 cells and detected an accumulation in γH2AX after cisplatin treatment or a cisplatin pre-treatment and CEP-18770 combination treatment. Importantly, we found that increasing concentrations of cisplatin induced increased γH2AX levels. However, the cisplatin pre-treatment and CEP-18770 combination induced a stronger accumulation of γH2AX in the nucleus of DAOY cells. We measured Annexin V/PI by FACS, and we validated that the combination of cisplatin and CEP-18770 in DAOY cells induced apoptosis. We started to validate some of our data in MB primary cells, ICb1299, which also express p73. Increasing concentrations of etoposide reduced cell viability, and the combination of etoposide pre-treatment and CEP-18770 showed a stronger effect. Therefore, these data are in line with our previous experiments.

This study has provided us with insights into the treatment conditions that can induce cell death in MBs cell lines. We concluded that CEP-18770 has a significant effect in MBs cells. This must be validated in MBs primary cells, to evaluate the efficacy of the combination therapy. We worked with small doses, as we hypothesized that the effect of the drugs would be greater in MBs primary cells. However, more experiments are required to test this hypothesis. In this study, we demonstrated that CEP-18770 alone induces apoptosis of MB cell lines and has a synergistic effect with cisplatin, thus providing a solid foundation for future research.

## Data Availability

Data is contained within the article and Supplementary Material.

## Supplementary Materials

The following supporting information can be downloaded at https://www.mdpi.com/article/10.3390/pharmaceutics16050672/s1.
