# Supplementary material for: The Proteasome Inhibitor CEP-18770 Induces Cell Death in Medulloblastoma
Source: Pharmaceutics. 2024 May 16;16(5):672. doi: 10.3390/pharmaceutics16050672 (PMC11124782; doi:10.3390/pharmaceutics16050672)
Supplement: Supplementary file 1 [file pharmaceutics-16-00672-s001.zip › pharmaceutics-2948648-supplementary.docx]

Supplementary Figure S1. Proteasome Inhibitors reduced viability of aggressive and non-aggressive MBs cells. Representative IC50 fitting curves of CEP-18770 (A) and NPI-0052 (B) in MBs cells. DAOY, UW228-2, D-425 and D-458 cells were treated for 48 hours with different concentrations of CEP-18770 (A) and NPI-0052 (B) and viability was measured using Cell TiterGlo assay.

Supplementary Figure S2. Chemotherapy drugs, cisplatin and etoposide, reduce DAOY cells viability. DAOY and UW228-2 cells were treated for 48 hours with different concentrations of cisplatin (A) and etoposide (B) and viability was measured using Cell TiterGlo assay. C, D) DAOY cells were treated with two siRNA against p73 (sip73*3, sip73*5) for 48 h. Cells were collected and a western blot of lysed cells for p73 and GAPDH was performed. D) Cell viability analysis in scramble, sip73*1 and sip73*2 DAOY cells after treatment with different concentration of cisplatin for 24 h. *P  < 0.05, **P  < 0.001; ***P  < 0.0001.

Supplementary Figure S3. CEP-18770 induced cell death of MBs spheroids. DAOY and UW228-2 cells were grown as spheroids and after 4 days cells were treated with CEP-18770, cisplatin or a combination of CEP-18770 plus cisplatin. Cell medium was changed every 2 or 3 days and fresh 0.1 or 1 pM CEP-18770 or 1.98 nM cisplatin was added. A, B) The size of the spheroid was measured every 2 or 3 days and the data was quantified for DAOY (A) and UW228-2 (B). *p<0.05; **p<0.01; ***p<0.001.
