# Supplementary figures and images for: The Proteasome Inhibitor CEP-18770 Induces Cell Death in Medulloblastoma
Source: Pharmaceutics. 2024 May 16;16(5):672. doi: 10.3390/pharmaceutics16050672 (PMC11124782; doi:10.3390/pharmaceutics16050672)

## Slide 1
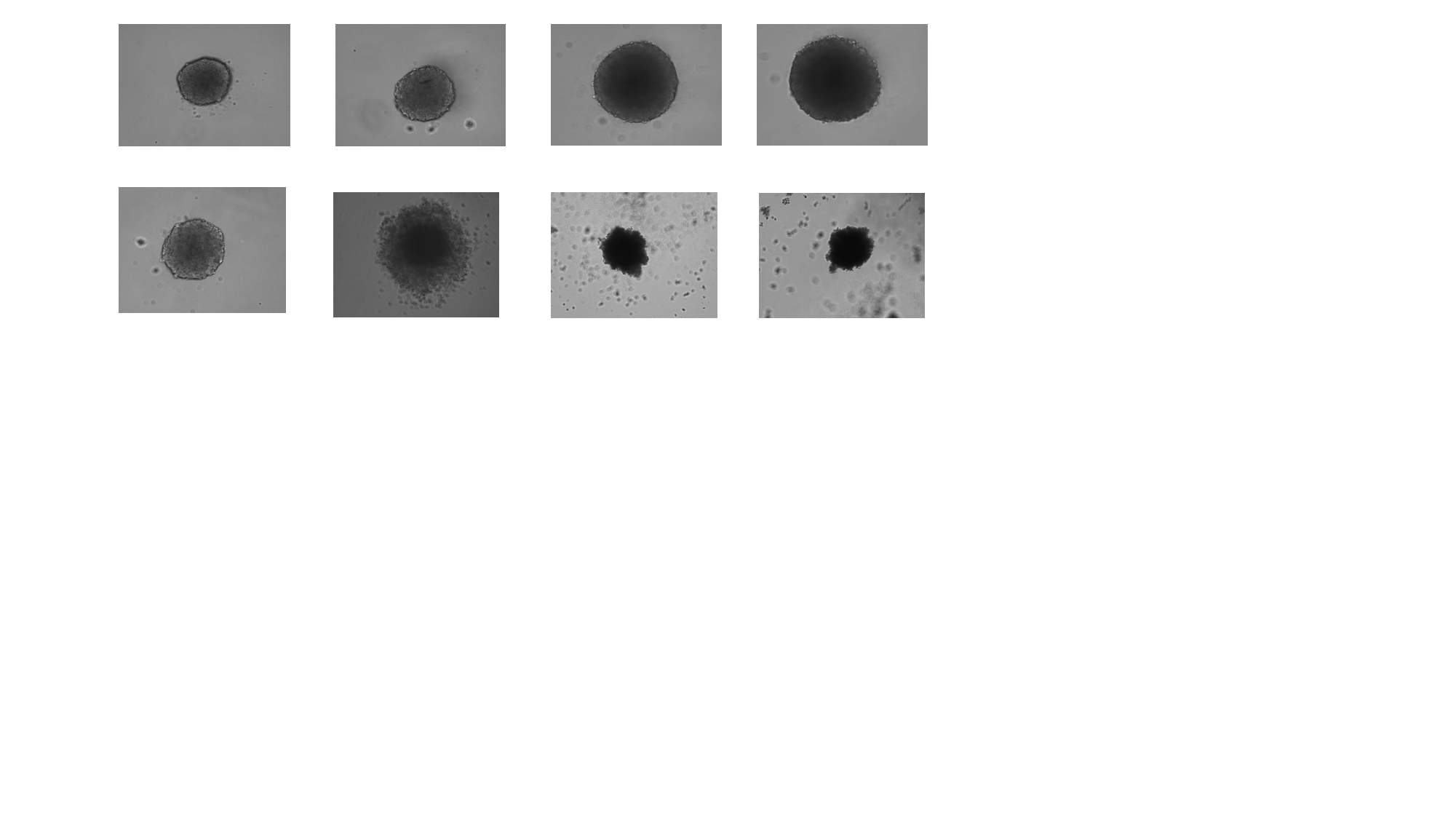

Supplement: Supplementary file 1 [file pharmaceutics-16-00672-s001.zip › pharmaceutics-2948648-Supplementary Figure S1.pptx]

## Slide 1
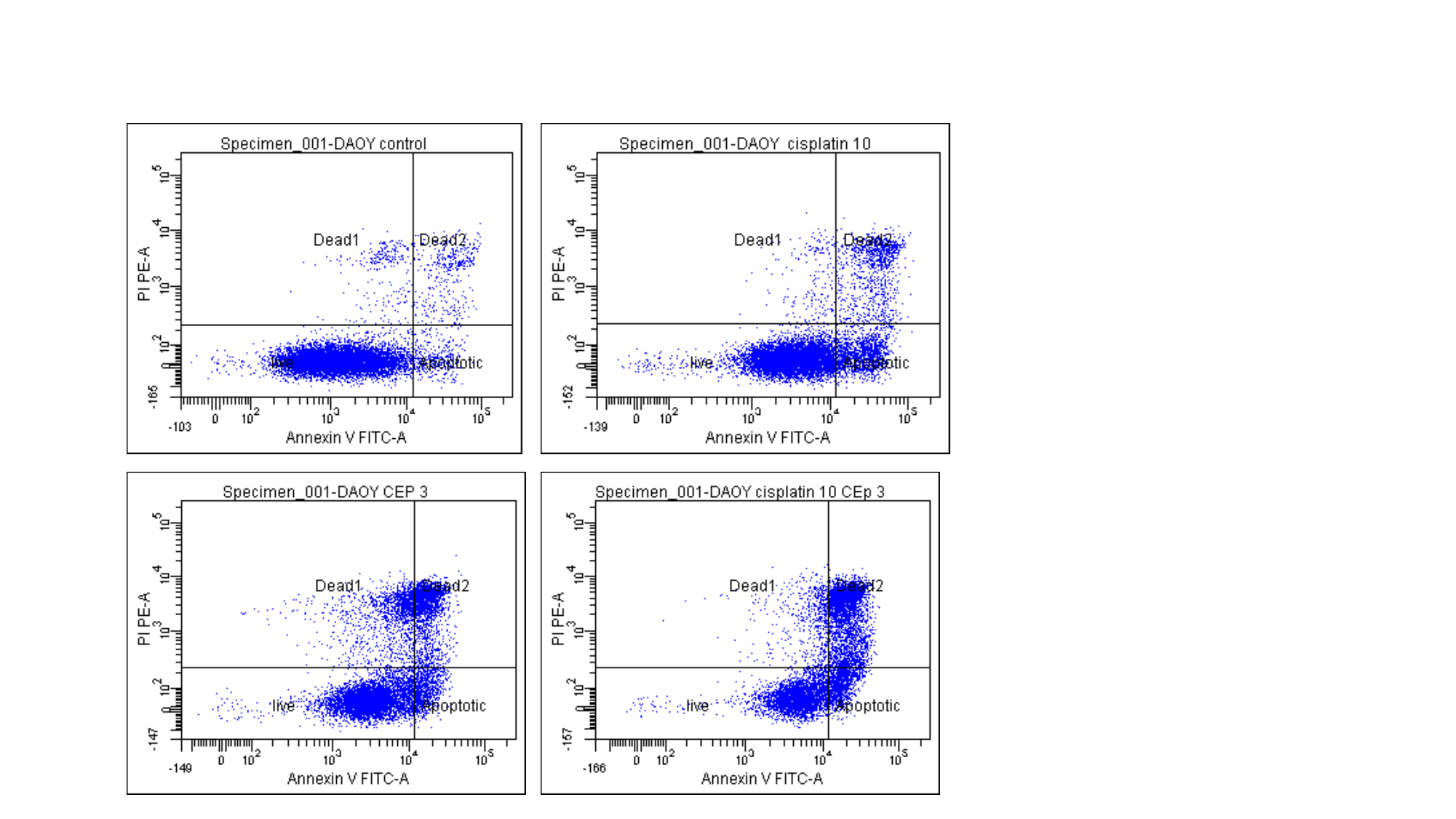

## Slide 2
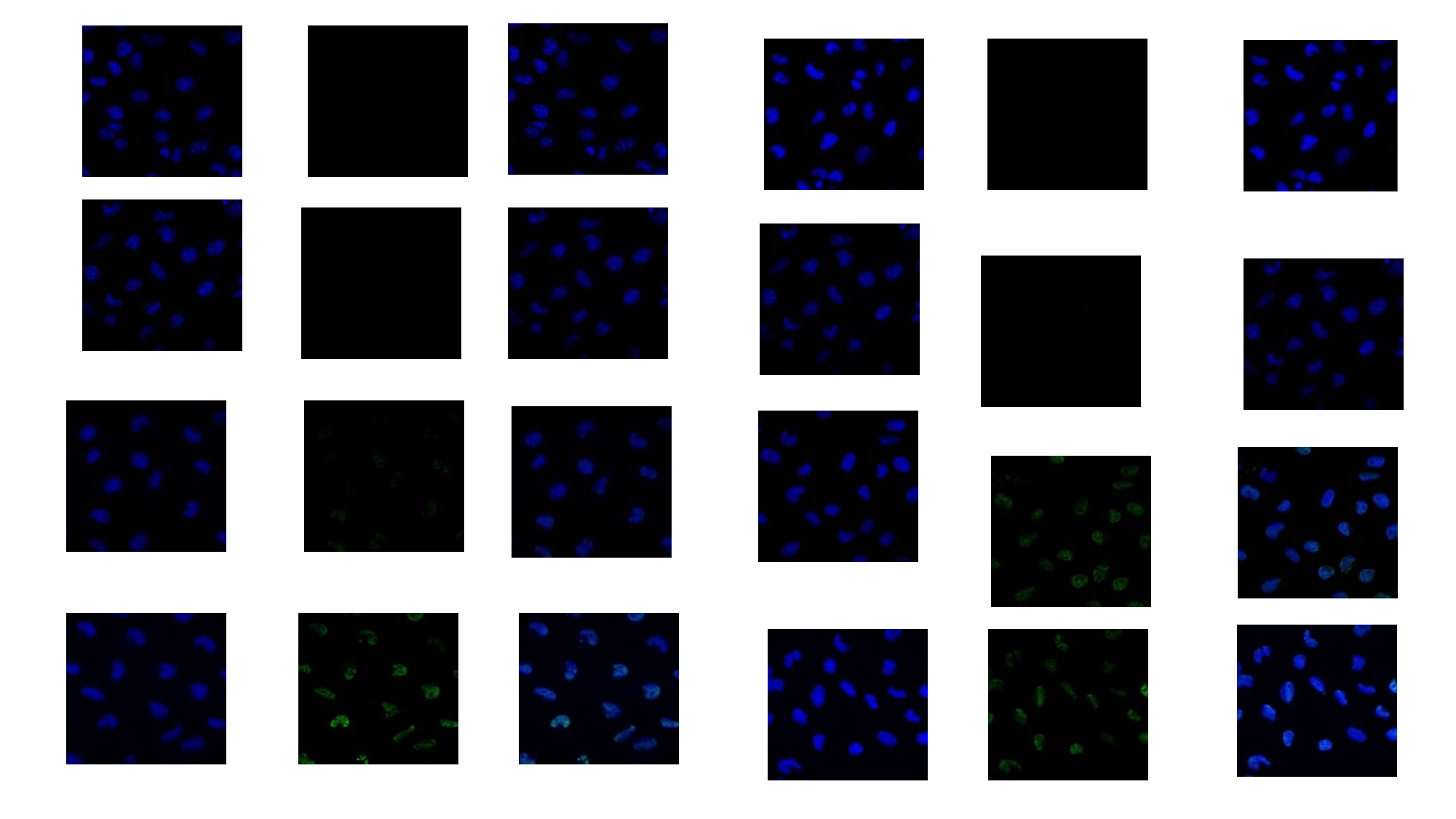

## Slide 3
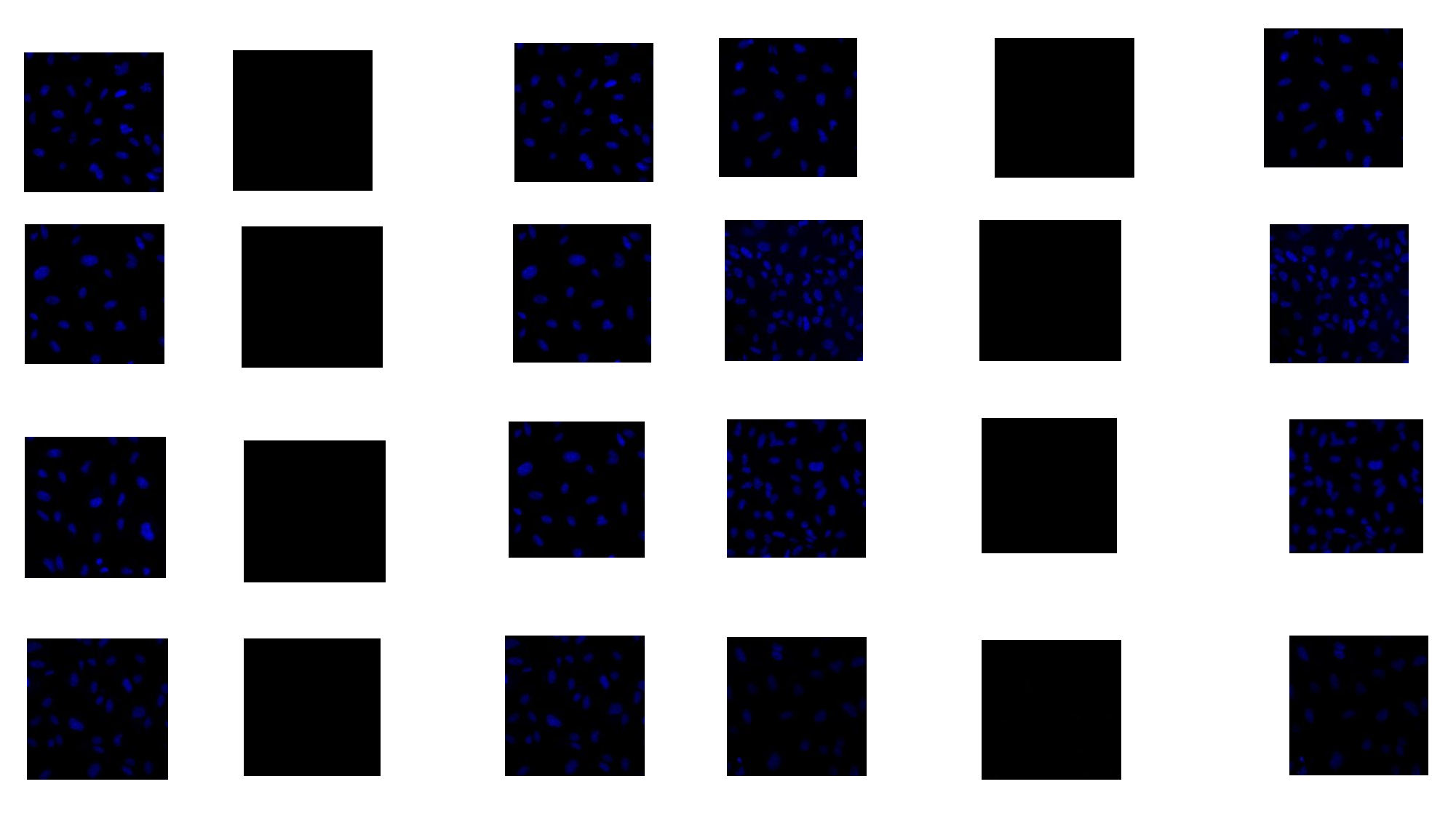

## Slide 4
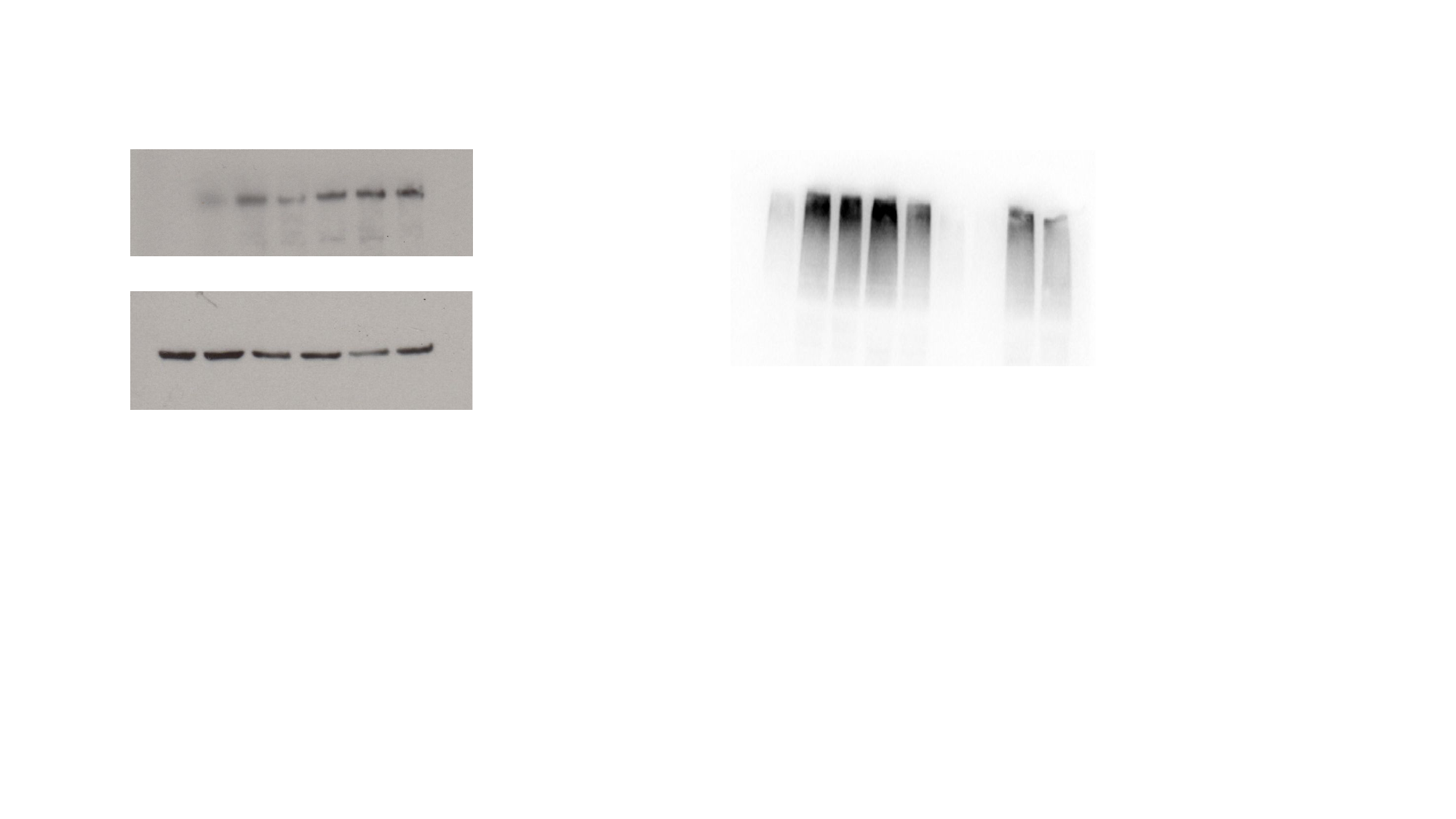

Supplement: Supplementary file 1 [file pharmaceutics-16-00672-s001.zip › pharmaceutics-2948648-Supplementary Figure S2.pptx]

## Slide 1
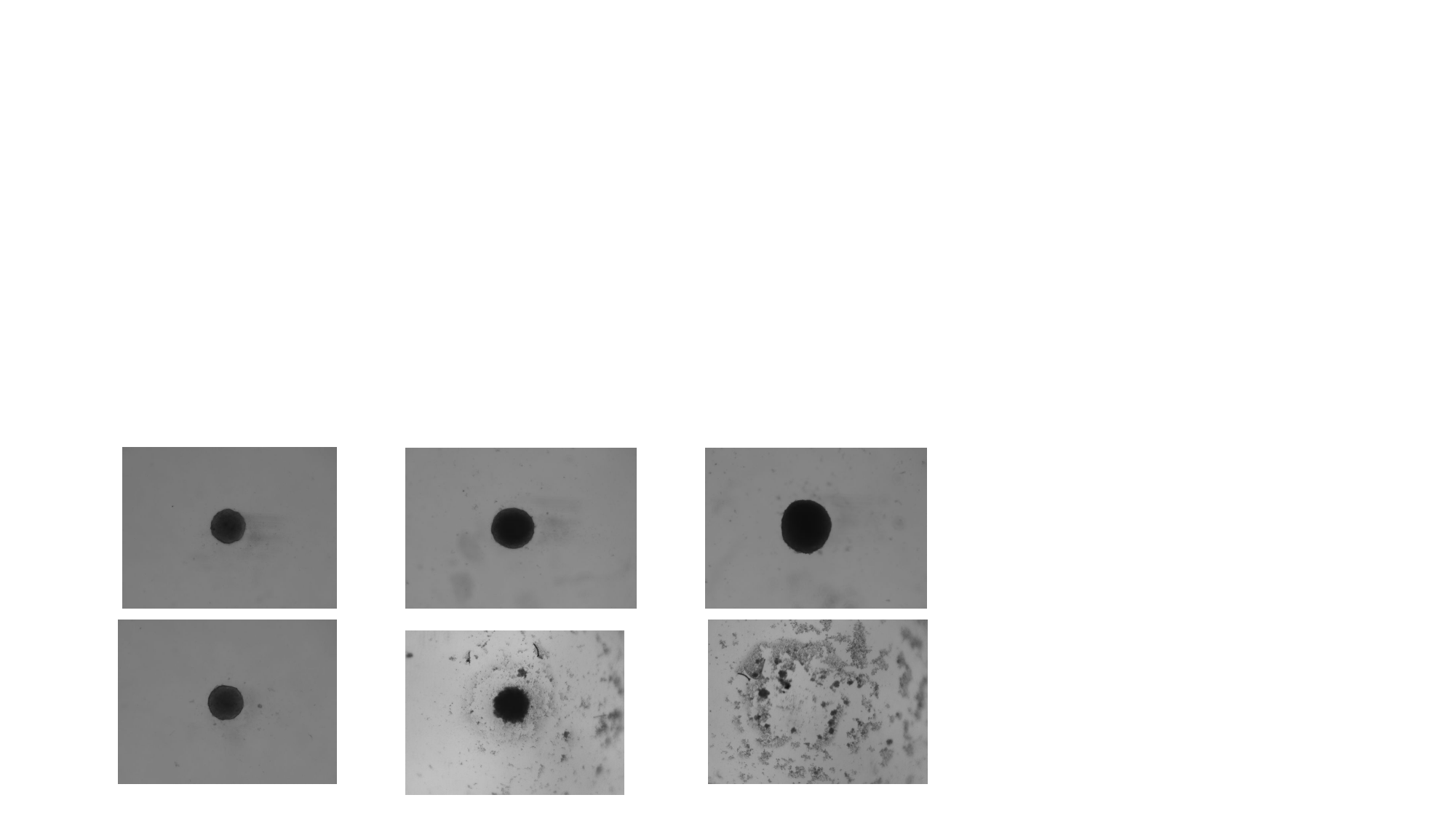

Supplement: Supplementary file 1 [file pharmaceutics-16-00672-s001.zip › pharmaceutics-2948648-Supplementary Figure S3.pptx]
